# Drones and Workers of Honeybee *Apis mellifera* L. Dried Powder: Chemical Composition, Antioxidant, and Anticancer Assessment

**DOI:** 10.1002/fsn3.4581

**Published:** 2024-11-07

**Authors:** Tarek Gamal Abedelmaksoud, Mohamed Salah El‐Masarawy, Ammar B. Altemimi, Mohammad Ali Hesarinejad, Ahmed M. S. Hussein, Sayed Saad Smuda

**Affiliations:** ^1^ Food Science Department, Faculty of Agriculture Cairo University Giza Egypt; ^2^ Department of Economic Entomology and Pesticides, Faculty of Agriculture Cairo University Giza Egypt; ^3^ Department of Food Science, College of Agriculture University of Basrah Basrah Iraq; ^4^ College of Medicine University of Warith Al‐Anbiyaa Karbala Iraq; ^5^ Research Institute of Food Science and Technology (RIFST) Mashhad Iran; ^6^ Food Technology Department, Food Industries and Nutrition Research Institute National Research Centre Dokki Giza Egypt

**Keywords:** anticancer, antioxidant, drones, honeybee, workers

## Abstract

This study explores honeybee as a food source through chemical analysis of pupa and adult stages of honeybee drones and workers (*Apis mellifera* L.). The findings reveal that drones exhibited higher protein and fat content, while workers have the highest carbohydrate levels. Additionally, seventeen amino acids and nine minerals were identified, with drones in the adult stage having elevated levels of fifteen amino acids, notably glutamic acid at 7.97 g/100 g. Moreover, 24 fatty acids were discovered, with oleic acid being most abundant in drones (55.96% in adult, 44.14% in pupa). Furthermore, potassium and sodium were the dominant minerals in both drone and worker samples; however, adult drone had higher concentrations of potassium, iron, copper, and phosphorus than the other samples. Also, nine phenolic compounds were identified, which exhibited the highest concentration in the adult drone sample (20095.72 μg/100 g). Importantly, the adult drone sample demonstrated significant cytotoxic effects on breast and colorectal cancer cells (MCF_7_ and HCT_116_), inhibiting them at rates of 98.1% and 81.5%, respectively. These results emphasize the nutritional potential of honeybees (especially adult drones) as a functional food, rich in amino acids, fatty acids and possessing antioxidant and anticancer properties.

## Graphical abstract

This study explores honeybees as a food source through a chemical analysis of pupa and adult stages of honeybee drones and worker bees (*Apis mellifera* L.). Drones exhibited higher protein and fat content, while worker bees had the highest carbohydrates. Drone powder demonstrated significant cytotoxic effects on breast and colorectal cancer cells (MCF7 and HCT116) at 98.1% and 81.5%, respectively.

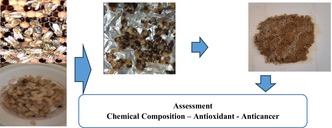

## 1 Introduction

Honeybee “*Apis mellifera* L.” is considered one of the most important and beneficial insects in the world. Honeybee products such as honey, pollen, royal jelly, wax, propolis, beebread, and bee venom have several beneficial effects like antimicrobial, antioxidant, therapeutic, and highly useful nutritional impacts (El‐Masarawy, El‐Bendary, and El‐Helaly 2021; Elsayed et al. 2021). Also, their nutritional as well as medicinal properties have been known for thousands of years and are mentioned in the Bible and Quran (Wehbe et al. 2019). Whereas, less well appreciated are the bees themselves compared with their products. Recently, food processing sustainability has been focused on 3 main axes as follows: (1) waste minimization, (2) future of food, and (3) bioactive compounds from natural sources. The world's population is rising, and food is scarce. In different countries, food consumption depends on habits, traditions, culture, ethnicity, and religion. So, meat became prominent in the human diet, and many people rely on animal protein (chicken, buffalo, cattle, fish, etc.) as their main source of protein. Recent debates have centered on how to meet future demand for meat‐based protein products (Talwar et al. 2024). In light of many environmental problems such as water resource availability, desertification, soil erosion, and climate change, it became clear and necessary to find alternative sources of protein to bridge the gap of demand (Smith et al. 2020).

Recently, insects have garnered global attention (worldwide, about 113 nations eat 2000 bug species) as a future protein source because they are highly nutritious, can be raised with few resources, and have a smaller environmental impact than traditional domesticated and farm animals (Li et al. 2023; Mohsen et al. 2024). Insects can improve global food systems by changing food sources. Whole insects may be eaten and handled in familiar ways, making them more consumer‐friendly. People commonly consume insects in various stages including eggs, larvae, pupae, nymphs, and adults. Moreover, beetles, caterpillars, ants, bees, wasps, grasshoppers, locusts, true bugs, aphids, leafhoppers, termites, and flies are the most commonly eaten species (Poma et al. 2017). In America and Europe, which do not eat insects, people can mix insect protein extracted from protein or insect‐dried powder with any food product to make it functional.

Many insects contain protein, fat, vitamins, and minerals (Li et al. 2023). Additionally, some researchers found that common edible insects had 9%–25% protein compared to 15%–22% in meat (Ghaly and Alkoaik 2009). Insects may carry 36.5% plant protein. One of the most promising alternative foods is “honeybee brood,” especially drones, which have a high nutritional value and are rich in vitamins and antioxidants. The global production of honeybee brood (*Apis mellifera* L.) makes it a food source. To minimize varroa mite (varroosis) “*Varroa destructor*,” the most damaging parasite affecting honeybees worldwide, many apiaries adopt drone brood removal. Free drone brood provides beekeepers with farmed insects (Jensen et al. 2019). In 2015, EFSA researched insect farming and eating, excluding honeybee production because Europe is not closed (EFSA, 2015). Recently, honeybee larvae, pupae and adults have been used in food as a proteinsource because apiaries produce bee brood in appropriate quantities, especially brood of drones (Ghosh, Jung, and Meyer‐Rochow 2016; Ghosh et al. 2020). Additionally, lyophilized bee larvae reduced cholesterol and triglycerides in experimental rats. Moreover, it protects liver functions, boosts the immune system, and can be used as animal feed (Açikgöz and Yücel, 2016).

The consideration of honeybee brood as an alternative or supplementary food is suffering from a dearth of scientific research on it. So, this work focused on honeybee *Apis mellifera* L. (pupae and adults of drones and workers) to shine a spotlight on the nutritional value and therapeutic activity of honeybees themselves. Therefore, this work aims to investigate the prospects of pupae and adults of drones and workers as new patterns for human nutrition and food sustainability systems in the world. Moreover, the study investigates the chemical composition, antioxidant capacity, and anticancer properties of these developmental stages of honeybee after being processed into powder form.

## 2 Materials and Methods

### 2.1 Chemicals

All reagents and chemicals used in this research were of analytical grade. Gallic acid, Folin–Ciocalteu reagent, 2,2‐ diphenyl‐1‐picrylhydrazyl (DPPH), 3‐(4,5‐dimethylthiazol‐2‐yl)‐2,5‐diphenyltetrazolium bromide (MTT), ethanol, sodium bicarbonate, sodium acetate, and butylated hydroxytoluene (BHT) were obtained from Sigma Chemical Co. Ltd. (St. Louis, MO, USA).

### 2.2 Collection of Honeybee Individual Samples (Pupae and Adults of Drone and Worker)

Pupa and adult drones and workers of *Apis mellifera* L. were collected from the apiary in the Faculty of Agriculture, Cairo University, Egypt. All four samples of honeybee (drone pupa “DP,” worker pupa “WP,” drone adult “DA,” and worker adult “WA”) were dried in an oven (Shel‐Lab, Cornelius, OR, USA) at 40°C for 24 h until no further weight reduction was measurable, then subjected to grinding by an analytical mill to a size of 1 mm (Cole‐Parmer, Vernon Hills, IL, USA), sieved up to 50 mesh, and kept in the dark place at room temperature for analysis.

### 2.3 Chemical Composition of Honeybee Powder Samples

Proximate analysis, including protein, lipids, moisture, and ash, was determined following standard methodology (AOAC 2016). Total carbohydrates were estimated by difference, and the energy was estimated as per the following equation (Ganogpichayagrai and Suksaard 2020).

$$\text{Energy}\;\left(\text{kcal}\right)=4\times \left(g\;\text{protein}+g\;\text{carbohydrates}\right)+9\times \left(g\;\mathrm{fat}\right)$$

### 2.4 Minerals Composition

Minerals content of honeybee samples was examined using inductively coupled plasma‐atomic emission spectroscopy (ICP‐OES: Icap6000 serious, Serial No. Icp‐20,080,614, England). The examination was performed in duplicate and determined the following microelements: Ca, Mg, Na, K, Fe, Zn, Cu, Mn, and P.

### 2.5 Color values

Color parameters (L*, a*, and b*) were measured using a Minolta colorimeter (Model CR‐400, Konica Minolta, Inc., Tokyo, Japan). The L* value indicates lightness, while the a* value represents redness (positive values) and greenness (negative values). The b* value denotes yellowness (positive values) and blueness (negative values).

### 2.6 Bioactive Profile

The phenolic acids in honeybee samples were extracted and analyzed following the method described by Kim et al. (2006). In summary, 1 g of each sample was placed in a fast‐fit conical flask. Then, 20 mL of a 2 M solution of sodium hydroxide were poured into the flasks. The flasks were then flushed with nitrogen gas and the stoppers were inserted. Following a 4‐h agitation of the flasks at ambient temperature, the pH was further lowered to 2 using 6 M hydrochloric acid. Each flask's contents were subjected to centrifugation at a speed of 3075 g for 10 min, and the resulting liquid above the sediment was gathered. The phenolic components were extracted using a mixture of 50 mL ethyl ether and ethyl acetate in a 1:1 ratio, and this extraction process was repeated twice. The organic phase was isolated, and the solvent was removed using evaporation at a temperature of 45°C. The remaining substances were then dissolved again in 2 mL of methanol and subjected to analysis using HPLC. The HPLC apparatus and the analytical column used for tocopherols were identical. The phenolic acids and flavonoids were identified and calibrated through a process of comparing them with established standards.

The total phenolic content was determined according to the Folin–Ciocalteu procedure (Abedelmaksoud et al. 2018). The sample (100 μL) was oxidized with Folin‐Ciocalteau reagent (250 μL). The absorbance was measured at 765 nm. The total phenolic content was determined using the gallic acid calibration curve and reported as micrograms of gallic acid equivalent (μg GAE) per gram of sample.

The total flavonoid content was determined according to Mohamed et al. (2021) with some modifications. Briefly, 250 μL of 5% NaNO_2_ was mixed with 500 μL of extract. After 6 min, 2.5 mL of a 10% AlCl_3_ solution was added. After 7 min, 1.25 mL of 1 M NaOH was added, and the mixture was centrifuged at 5000 *g* for 10 min. The absorbance of the supernatant was measured at 510 nm against the solvent blank. The total flavonoid content was expressed as mg of catechin equivalent (CE) per g of sample.

Free radical scavenging capacity was assessed using DPPH (Elsayed et al. 2022). Briefly, the sample (50 μL) was mixed with the DPPH solution (3.95 mL) for 1 h in the dark. The absorbance was measured at 517 nm. Percent inhibition of the DPPH free radical was calculated by Equation (1):

(1) $$\text{Inhibition}\;\left(\%\right)=100–\left[\left(A\;\text{control}–A\;\text{sample}\right)/A\;\text{control}\right]$$

where *A* control is the absorbance of the control (reagent without the tested sample). A sample is the absorbance of the tested sample. Trolox (within a concentration range from 10 to 150 μmol/L) was used to prepare the standard curve. Results were expressed as μg Trolox equivalents (TE)/g sample.

A ferric‐reducing antioxidant power (FRAP) assay was conducted based on Benzie and Choi (2014). The FRAP reagent was prepared daily and warmed to 37°C in a water bath before use. One hundred microliters of the extracts were added to 3 mL of the FRAP reagent. The absorbance of the reaction mixture was then measured at 593 nm after 4 min at room temperature. The standard curve was carried out using FeSO_4_ solution, and the results were expressed as μmol Fe (II)/g dry weight of plant material.

### 2.7 Cytotoxic Effect on Human Cell Lines

Mitochondrial‐dependent reduction of yellow MTT (3‐(4,5‐dimethylthiazol‐2‐yl)‐2,5‐diphenyl tetrazolium bromide) to purple formazan was used to determine cell viability. All procedures were performed in a sterile environment using a biosafety class II laminar flow cabinet (Baker, SG403INT, Stanford, ME, USA). Cells were suspended in DMEM‐F12 medium [for MCF_7_ (Human Caucasian breast adenocarcinoma) and HCT_116_(Human colorectal carcinoma)] alongside a normal cell line (BJ1), a 1% antibiotic‐antimycotic mixture (10,000 U/mL Potassium Penicillin, 10,000 μg/mL Streptomycin Sulfate, and 25 μg/mL Amphotericin B), and 1% l‐glutamine at 37°C under 5% CO_2_. Cells were cultured in batches for 10 days, then seeded at a concentration of 10 × 10^3^ cells/well in fresh complete growth medium in 96‐well microtiter plastic plates at 37°C for 24 h under 5% CO_2_ using a water‐jacketed carbon dioxide incubator (Sheldon, TC2323, Cornelius, OR, USA). Media was aspirated, fresh medium (without serum) was added, and cells were incubated either alone (negative control) or with different concentrations of sample to give a final concentration of (100, 50, 25, 12.5, 6.25, 3.125, 0.78, and 1.56 μg/mL). After 48 h of incubation, the medium was aspirated, and 40 μL of MTT salt (2.5 μg/mL) was added to each well and incubated for a further 4 h at 37°C under 5% CO_2_. To stop the reaction and dissolve the formed crystals, 200 μL of 10% sodium dodecyl sulfate (SDS) in deionized water was added to each well and incubated overnight at 37°C. A positive control composed of 100 μg/mL was used as a known cytotoxic natural agent that gives 100% lethality under the same conditions (El‐Menshawi et al. 2010). The absorbance was then measured using a microplate multi‐well reader (Bio‐Rad Laboratories Inc., model 3350, Hercules, CA, USA) at 595 nm and 620 nm as a reference. The statistical significance between samples and the negative control (vehicle‐containing cells) was evaluated using an independent SPSS 11 *t*‐test. The solvent used to dissolve extracts was DMSO, and its final concentration on the cells was < 0.2%. According to the following formula, the percentage of change in viability was calculated: ((reading of extract/reading of negative control) − 1) × 100. Using the SPSS 11 program, the IC_50_ and IC_90_ were determined using probit analysis. The degree of selectivity of the synthetic compounds was expressed as SI = IC_50_ of the pure compound in a normal cell line/IC_50_ of the same pure compound in a cancer cell line, where IC_50_ is the concentration required to kill 50% of a cell population.

### 2.8 Fatty Acids Profile

The fatty acid composition was determined by the conversion of oil to fatty acid methyl esters prepared by adding 1.0 mL of n‐hexane to 15 mg of oil, followed by 1.0 mL of sodium methoxide (0.4 mol) (Zahran and Tawfeuk 2019). The mixtures were vortexed for 30 s and were allowed to settle for 15 min. The upper phase containing the FAMEs was recovered and analyzed by gas chromatography (GC‐FID). Condition of GC Analysis: Type of GC: Perkin Elmer Auto System XL; Equipped with flame ionization detector (FID); fused silica capillary column ZB‐Wax (60 m × 0.32 mm i.d.); Oven temperature was maintained initially at 50°C, then programmed from 50**°**C to 220°C, at rate 4°C/min.; Injector temperature Was 250°C. Detector temp. 250°C. Carrier gas: Helium, flow rate 1 mL/min.

### 2.9 Amino Acids Profile

Amino acid profiles were analyzed as per the following protocol: HPLC analysis was carried out using an Agilent 1260 series. The separation was carried out using an Eclipse Plus C_18_ column (4.6 mm × 250 mm i.d., 5 μm). The mobile phase consisted of buffer (sodium phosphate dibasic and sodium borate), pH 8.2 (A), and ACN:MeOH:H_2_O 45:45:10 (B) at a flow rate of 1.5 mL/min.

### 2.10 Statistical Analysis

Experimental results were analyzed using analysis of variance (ANOVA) XLSTAT software version 2014, 5.03 (Addinsoft, New York, NY, USA) in three repeats and expressed as the mean ± standard error of the mean. The significance of differences between sample means was calculated at *p*‐value ≤ 0.05 and was considered significant.

## 3 Results and Discussion

### 3.1 Chemical Composition of Pupa and Adult for Drones and Workers of Honeybee *A*. *Mellifera* L.

Table 1 concluded that protein content increased and was associated with developmental stages from pupae to adults. Adult drones had the highest protein content at 61.71%, followed by adult workers at 53.53%. Also, for the pupal stage, the values of protein decreased to 49.45% in drones and to 45.45% in honeybee workers. Conversely, honeybee fat content decreased during developmental stages. The largest fat value was observed in the pupae of drones (20.02%), followed by the pupaeof workers (14.80%). On the other hand, fat values decreased deeply to 7.35% and 7.08% for adult drones and workers, respectively.

**TABLE 1:** Chemical composition of pupa and adult for drones and workers of *A*. *mellifera* L.

Sample	Protein (%)	Fat (%)	Carbohydrates (%)	Moisture (%)	Ash (%)	Energy (kcal)
Drone (pupa)	49.45 ± 0.14c	20.02 ± 0.05a	16.00 ± 0.37c	6.85 ± 0.22d	7.70 ± 0.01c	441.98
Worker (pupa)	45.45 ± 0.24d	14.80 ± 0.05b	21.73 ± 0.49a	9.14 ± 0.13b	8.88 ± 0.01b	401.92
Drone (adult)	61.71 ± 0.19a	7.35 ± 0.16c	14.44 ± 0.21d	8.27 ± 0.15c	8.23 ± 0.18bc	370.75
Worker (adult)	53.53 ± 0.17b	7.08 ± 0.12c	19.43 ± 0.72b	10.43 ± 0.16a	9.53 ± 0.09a	355.56

*Note:* The experimental values (means and SD for *n* = 3) with small letter are significantly different (*p* ≤ 0.05).

In the same line, Ghosh, Jung, and Meyer‐Rochow (2016), found that honeybee (*Apismelliferaligustica*) larvae, pupae, and adults had high protein levels of 35.3%, 45.9%, and 51.0%, respectively. Moreover, they reported that fat content dropped from 16.0 in pupae to 6.9% in adult workers. However, Makkar et al. (2014) also mentioned that the fat content in larvae and pupae of honeybees from Mexico ranged between 19.0% and 20%, respectively. Furthermore, our results of protein at the pupal stage were almost identical to those reported by Ramos‐Elorduy (2005) and Raheem et al. (2019), who detected about 49.0% of protein in pupae. Moreover, Finke (2005) estimated the protein content of 94 g per kg of bee brood to be about 40.5% on a dry matter basis, and he determined the fat content for honeybee brood to be 20.3% based on dry matter. On the other hand, Kim et al. (2018) found that protein content in drone pupae was 42.9% on dry matter. In contrast, Rutka et al. (2021) found that the honeybee larval and pupae homogenate had the following chemical structure: protein 35.3% and 45.9%, fat 14.5% and 16.0%, carbohydrates 46.1% and 34.3%, and ash 4.1% and 3.8%, respectively. Data in Table 1 pertains to honeybee pupae, which have more fat than adults. Drone pupae have three times the fat of adult drones. Whereas, worker pupae are twice as fat as adult workers. Furthermore, pupae and adults of honeybee workers had the highest carbohydrate content, 21.73% and 19.43%, respectively. Drone carbohydrate content fell from 16.00% in the pupal stage to 14.44% in the adult stage. These results matched those of Ghosh, Jung, and Meyer‐Rochow (2016) on pupae and worker adult carbohydrate content, in which the carbohydrate proportion dropped from 34.3% in pupae to 30.6% in adults. Additionally, Sawczuk et al. (2022) found drone homogenate to be a significant carbohydrate source. Table 1 also shows that adult and pupal worker ash proportions were highest at 9.53% and 8.88%. According to Ghosh, Jung, and Meyer‐Rochow (2016), adult worker ash concentration was 11.52%, the highest among larval and pupae worker ash levels of 4.1% and 3.8%, respectively. Additionally, pupae and adult drones had the lowest ash values at 7.70% and 8.23%. Nowak et al. (2016) and Choi (2021) found mature drone and worker ash levels of 4.9%–6.0% dry weight.

### 3.2 Amino Acids Composition of Pupa and Adult for Drones and Workers of *A*. *Mellifera*

Table 2 shows that adult drones had the greatest values of most total amino acids, except for proline in adult workers and tyrosine in drone pupae. Overall, glutamic acid is the highest amino acid (essential and non‐essential) with amount reached to 7.97 and 6.09 g/100 g in drone adults and pupae, respectively. According to Ghosh, Jung, and Meyer‐Rochow (2016), the highest amino acid was glutamic acid, with 8.4 and 6.0 g/100 g in pupae and adults of honeybee workers, respectively.

**TABLE 2:** Amino acid composition (g/100 g DM and % of total amino acids) of pupa and adults for drones and workers of *A*. *mellifera*.

Amino Acids	Drone (pupa)		Worker (pupa)		Drone (adult)		Worker (adult)	
	g	%	g	%	g	%	g	%
Aspartic	2.84	7.09	2.95	9.22	3.66	7.71	2.84	8.02
Glutamic	5.79	15.56	6.09	18.45	7.97	16.49	5.20	13.43
Serine	1.66	4.15	1.38	4.30	2.12	4.47	1.79	5.05
Histidine	0.58	1.46	0.54	1.69	0.68	1.43	0.47	1.33
Glycine	2.39	5.97	1.64	5.12	2.91	6.13	2.36	6.66
Threonine	1.43	3.57	1.30	4.06	1.97	4.15	1.51	4.26
Proline	2.75	7.59	2.76	8.63	3.16	6.65	5.07	13.44
Tyrosine	1.65	4.12	1.48	4.63	1.55	3.27	1.09	3.08
Cystine	0.45	1.12	0.32	1.00	0.52	1.09	0.40	1.13
Valine	2.16	5.39	1.84	5.75	2.63	5.54	2.11	5.96
Methionine	0.78	1.94	0.60	1.88	0.88	1.85	0.59	1.67
Phenylalanine	1.31	3.27	1.28	4.00	1.64	3.45	1.20	3.39
Isoleucine	1.80	4.50	1.59	4.97	2.30	4.84	1.77	5.00
Leucine	3.00	7.50	2.64	8.25	4.01	8.44	3.06	8.64
Lysine	2.52	6.29	2.31	7.22	3.39	7.14	2.49	7.03
Alanine	2.90	7.24	2.00	6.25	3.93	8.29	2.96	8.36
Arginine	3.21	8.02	2.28	7.12	5.01	10.58	3.82	10.79
Total	37.22	100%	33.00	100%	48.33	100%	38.73	100%

Table 2 shows that eight necessary amino acids were discovered, but tryptophan was not detected. However, leucine was the biggest essential amino acid in pupae and adults of drones, with 4.01 g/100 g in adult drones and 3.00 g/100 g in pupae drones. As with most edible insects, such as *Parachartegus apicalis* (Hymenoptera), *Brachygastra azteca* (Hymenoptera) (Yen 2009), *Rhynchophorus phoenicis* larvae (Coleoptera) (Ekpo and Onigbinde 2005), and *Heliothis armigera* (Lepidoptera) (Ghaly and Alkoaik 2010). Moreover, lysine was the second most abundant essential amino acid, followed by valine as the third, in both adult and pupal drones. Adult drones contained the highest levels of lysine and valine, with 3.39 g/100 g and 2.63 g/100 g, respectively. As well as, in drone pupae, the lysine and valine contents were 2.52 g/100 g and 2.16 g/100 g, respectively.

Proline content was 5.07 g/100 g and 3.16 g/100 g for workers and drones at the adult stage, respectively. Ghosh, Jung, and Meyer‐Rochow (2016) found no proline in honeybee workers. Otherwise, Ghosh et al. (2020) detected proline in pupae of Danish (Buckfast bees) and Korean (Italian bees) drones at 1.52 and 3.30 g/100 g. Moreover, proline was detected in Danish and Korean drone adults at 2.33 and 4.65 g/100 g, respectively. Our investigation generally supported Ghosh, Jung, and Meyer‐Rochow (2016) on Italian honeybee workers and Ghosh et al. (2020) on Danish (Buckfast) and Italian honeybee drones. Ghosh, Jung, and Meyer‐Rochow (2016) found that pupa and adult workers have 3.2 and 3.8 g/100 g of leucine, respectively, which is higher than other necessary amino acids. These results agree with our findings which were 2.64 and 3.06 g/100 g for pupae and adult workers. Ghosh et al. (2020) found that leucine was the most abundant essential amino acid in pupae of Danish and Korean drones, reaching 4.26 and 3.84 g/100 g. Also, adult drones of both bees had leucine levels of 5.51 and 5.60 g/100 g, while they were 3.00 and 4.01 g/100 g for pupae and adult drones in our study, respectively. Like us, Ghosh et al. (2020) found seventeen amino acids in Danish and Korean bees except tryptophan. Ghosh, Jung, and Meyer‐Rochow (2016) found that glutamic acid has the highest value of all amino acids (essential and non‐essential) at 8.4 and 6.0 g/100 g in pupae and adult workers. Ghosh et al. (2020) found 8.78 and 10.28 g/100 g in Danish and Korean drone pupae. Similar values in mature bee drones, 8.74 and 12.27 g/100 g (Ghosh et al. 2020). Interestingly, Ghosh, Jung, and Meyer‐Rochow (2016) identified tryptophan only in adult workers at traces (0.01 g/100 g).

Tables 1 and 2 indicate that both pups and adults of drones and workers exhibit high amino acid and protein content, aligning with findings from previous studies. Ghosh, Jung, and Meyer‐Rochow (2016) reported similar results for Italian honeybee workers, while Ghosh et al. (2020) observed comparable trends in Danish and Italian honeybee drones. In Tables 1 and 2, drone adults had the most protein (61.71%) and total amino acids (48.33 g/100 g DM) compared to other samples. This high protein content made honeybees a viable protein source, surpassing pork, beef, egg, and chicken, which had a protein content of 27.7%, 40.5%, 52.7%, and 54.7%, respectively (Ghosh et al. 2020). Bee brood and adults have higher nutritional value due to their high amino acid concentration (8 necessary amino acids). Bee proteins include practically all necessary amino acids (Ghosh, Jung, and Meyer‐Rochow 2016; Isidorov, Bakier, and Stocki 2016). The total amino acids in brood homogenate vary by honeybee caste, with 37.6%–40.6%, 35.1%–38.4%, and 35.6%–35.7% for drone, queen, and worker larvae, respectively, as reported by Lazaryan (2002). According to the same study, drones had 15.5%–16.3% essential amino acids, queens 18.9%–19.0%, and worker larvae homogenates 15.9%–17.0%. Their contents increased throughout larval growth and peaked in pupae (Hu and Li 2001; Ghosh, Jung, and Meyer‐Rochow 2016). Generally, the most abundant amino acids in honeybees are glutamic, aspartic, proline, valine, leucine, and lysine.

### 3.3 Fatty Acids Composition of Pupa and Adult for Drones and Workers of *A. Mellifera*

Honeybee brood and individuals appear to have a rich and varied supply of fatty acids, as shown in Table 3. Furthermore, pupa of drones had a much higher fat percentage of 20.02% compared to 14.80% for pupa of workers (Table 1). However, fatty acids vary substantially among pupa and adults in drones and workers. Generally, monounsaturated fatty acids predominated. Interestingly, oleic, palmitic, and stearic were the most abundant fatty acids, accounting for 93% in drone pupae and 91% in worker pupae, as well as 76% and 65% in drone and worker adults, respectively.

**TABLE 3:** Fatty acids composition of pupa and adult for drones and workers of honeybee *A*. *mellifera* L.

*N*	Fatty acids	Relative concentration (%)			
		Drone (pupa)	Worker (pupa)	Drone (adult)	Worker (aadult)
1	Lauric acid (C12:0) (SFA)	0.29	0.41	—	—
2	Myristic acid (C14:0) (SFA)	3.45	2.84	0.50	0.96
3	Palmitic acid (C16:0) (SFA)	37.76	32.33	10.55	17.73
4	Stearic acid (C18:0) (SFA)	11.01	14.00	9.43	8.40
5	Behenic acid (C22:0) (SFA)	0.12	0.17	—	1.07
6	Arachidic acid (C20:0) (SFA)	0.08	0.17	0.50	—
7	Caproic (Hexanoic) acid (C6:0) (SFA)	0.03	—	—	0.48
8	Capric (Decanoic) acid (C10:0) (SFA)	0.03	0.04	—	—
9	Pentadecanoic acid (C15:0) (SFA)	—	0.17	0.58	—
10	Heptadecanoic (Margaric) acid (C17:0) (SFA)	0.20	0.55	2.68	1.30
11	Oleic acid (C18:1n9c) (MUFA)	44.14	43.94	55.96	38.26
12	Palmitoleic acid (C16:1n7) (MUFA)	0.26	0.37	0.77	0.34
13	Palmitoleic acid (C16:1n9) (MUFA)	0.44	0.37	4.15	0.56
14	Myristoleic acid (C14:1) (MUFA)	0.02	0.03	—	—
15	Elaidic acid (C18:1n9t) (MUFA)	0.09	0.25	0.92	1.77
16	Cis‐10‐Heptadecenoic acid (C17:1) (MUFA)	0.06	0.09	1.60	1.21
17	Cis‐11‐Eicosenoic acid (C20:1) (MUFA)	0.88	1.38	3.85	6.94
18	Nervonic acid (C24:1) (MUFA)	0.08	0.20	1.02	2.89
19	Linoleic acid (C18:2n6c) (PUFA)	0.35	0.68	1.74	5.47
20	Linolelaidic acid (C18:2n6t) (PUFA)	0.06	0.18	0.78	5.04
21	γ‐Linolenic acid (C18:3n6) (PUFA)	—	—	2.36	—
22	Cis‐11,14‐Eicosadienoic acid (C 20:2) (MUFA)	0.33	0.75	0.79	0.94
23	Cis‐13.16‐Docosadienoic acid (C22:2) (PUFA)	0.07	0.14	—	—
24	Cis‐8,11,14‐eicosatrienoic acid (C20:3n6) (PUFA)	0.21	0.56	1.84	6.65

Abbreviations: MUFA, Monounsaturated fatty acids; PUFA, polyunsaturated fatty acids; SFA, saturated fatty acids.

Oleic acid (MUFA) was highest in adult drones at 55.96%, followed by pupae of drones and workers with percentages 44.14, 43.94, respectively. Also, the lowest proportion was 38.26% for adult workers. Remarkably, honeybee broods are nutritionally dense of oleic, surpassing eggs, conventional meat, and most edible oils (excluding olive and canola oil) (Ghosh, Jung, and Meyer‐Rochow 2016). Among the fatty acids, palmitic acid ranked second in terms of abundance. The pupa of drones had the highest concentration of palmitic acid (37.76%). This was followed by the pupa of workers, adult workers, and adult drones, with percentages of 32.33%, 17.73%, and 10.55%, respectively. Whereas, pupae workers had the highest content of stearic (SFA) with a value up to 14.00%, followed by drone pupae, adult drone, and adult workers with percentages of 11.01%, 9.43%, and 8.40%, respectively (Table 3). Our results also supported the findings of Ghosh, Jung, and Meyer‐Rochow (2016), who found that 47.6% and 45.2% oleic acid, 35.1% and 14.4% palmitic acid, and 12.6% and 9.3% stearic acid in pupa and adult workers of *A*. *mellifera ligustica*. On the same trend, oleic acid was the predominant of all fatty acids for the pupal stage of Danish and Korean drones, with percentages of 43.04% and 43.48%, respectively. Also, for the adult stage of Danish and Korean drones with percentages of 63.48% and 58.46%, respectively (Ghosh et al. 2020). On the other hand, drone pupae had the most myristic and palmitic acids with values of 3.45% and 37.76%, respectively. Moreover, drone adults had the highest levels of pentadecanoic, palmitoleic, heptadecanoic, cis‐10‐heptadecanoic, oleic, arachidic, and γ‐linolenic acids (only found in drone adults) by 0.58%, 0.77%, 4.15%, 2.68%, 1.60%, 55.96%, 0.50%, and 2.36%, respectively. Nevertheless, pupa and adult workers also have distinctive values of fatty acids; worker pupa excelled by slight differences from the other samples in each of the following fatty acids: capric acid, lauric acid, myristoleic acid, stearic acid, and cis‐13.16‐docosadienoic acid, with percentages of 0.04%, 0.41%, 0.03%, 14.00% and 0.14%, respectively. Furthermore, the results of worker adults showed an increase of some fatty acids than other samples such as caproic acid, elaidic acid, linoleic acid, linolelaidic acid, cis‐11‐ eicosenoic acid, cis‐11,14‐ eicosadienoic acid, behenic acid, cis‐8,11,14‐eicosatetraenoic acid, and nervonic acid with percentages 0.48, 1.77, 5.47, 5.04, 6.94, 0.94, 1.07, 6.65, and 2.89, respectively (Table 3).

Exceedingly well, our findings went in the same trend as the results of Ghosh, Jung, and Meyer‐Rochow (2016). They found seven fatty acids identical to ours (10 fatty acids they estimated) in pupae and adults of *A*. *mellifera ligustica* workers. However, their results indicated that oleic, palmitic, and stearic have the supreme values in worker pupae, with proportions of 47.6%, 35.1%, and 12.6%, respectively. Also, oleic, eicosenic, and palmitic were the highest proportions in worker adults, with values of 45.2%, 19.2%, and 14.4%, respectively. Furthermore, they found capric acid, lauric acid, myristic acid, and linoleic acid with percentages (0, 0.2%), (0.4, 0.3%), (2.9, 0.6%), and (0, 7.8%) for both pupae and adults of workers, respectively (Ghosh, Jung, and Meyer‐Rochow 2016). Considering our results, which related to the analysis of fatty acids for both drones and workers of honeybee *A*. *mellifera*, 24 distinctive fatty acids were identified. So, oleic acid is considered predominant in the MUFA group at 55.96%, and palmitic acid, followed by stearic acid, is considered predominant in the SFA group at 37.76% and 14.00%, respectively (Table 3).

Type and amount of fatty acids determine fat advantages and risks. Excess SFA causes heart attack and atherosclerosis. Palmitic, myristic, and lauric acids raised cholesterol, but myristic was strongest (Mensink 1993). From Table 3, drone pupa has the most content of palmitic and myristic at 37.76% and 3.45%, respectively. Only pupae of drone and worker had lauric acid at 0.29% and 0.41%, respectively. Our study indicated that drones and workers honeybees had appropriate myristic acid levels, similar to eggs (0.4%), conventional meat (2.9%), and edible oil (0.5%). Moreover, levels of palmitic in adults of drone and worker were 10.55 and 17.73%, lower than eggs (25.7%), traditional meat (24.6%), and nearly to edible oil (17.5%). In contrast, values of palmitic rose in pupae of drone and worker to 37.76% and 32.33%. Fortunately, stearic acid reduces LDL cholesterol (Mensink 2005). However, worker pupa had 14.00% stearic acid, greater than conventional meat (12.80%). Whereas, the value of stearic acid in drone pupa reached 11.01%, higher than egg (9.3%) and edible oil (e.g., canola, olive, and palm oil) (8.6%) (Ghosh, Jung, and Meyer‐Rochow 2016).

Bee brood has high lipid and fatty acid content; triacylglycerols, free fatty acids, sterols, diacylglycerols, phospholipids, and lipid phosphorus were all included in the list of lipid components of bee brood, according to Lazaryan and Sotnikova (2004) and Isidorov, Bakier, and Stocki (2016). Furthermore, Bogdanov (2011) found that fatty acid levels are 4 g/100 g of homogenate fresh bee brood. It mainly consists of (52% of SFA, 46% of MSFA, and 2% of PUSFA). At the same time, the superior fatty acids were palmitic, oleic, and stearic (Finke 2005; Ghosh, Jung, and Meyer‐Rochow 2016). Paradoxically, 10‐hydroxy decenoic acid (10‐HDA) hasn't been found in drone homogenate (Isidorov, Bakier, and Stocki 2016).

Ghosh et al. (2020) discovered that palmitic, stearic, and myristic represented about 55.20% and 52.77% of the total fatty acids of drone pupae of Danish and Korean honeybees. At the same time, it was observed that the aforementioned three fatty acids accounted for approximately 52.22% of the total fatty acids in drone pupae (Table 3). Whereas, this representation declined to about 20.48% in adult drones with clear superiority for palmitic and stearic with percentages of 10.55 and 9.43, respectively (Table 3). Furthermore, behenic and arachidic fatty acids came as the third highest ones for Danish and Korean drones with percentages of 2.24 and 3.44, respectively (Ghosh et al. 2020). However, our study discovered 2.68% margaric acid in adult drones, third after palmitic and stearic (Table 3). It is worth noting that saturated fatty acids, including lauric, myristic, and palmitic acids except stearic, raise cholesterol and are harmful to the human body. However, this study identified lauric acid in pupae of drones and workers with low traces at 0.29% and 0.41%, respectively, which is particularly promising, since adult drones and workers also lack it. Further, adult drones and workers had a low content of palmitic and myristic (Table 3). Mensink and Katan (1987) and Friedt and Snowdon (2010) found that monounsaturated fatty acids lower LDL, making diets healthier. Moreover, MUFA and PUFA are beneficial and improve cardioprotection, atherosclerosis, and disability. Whereas, SFA is linked to obesity and other body disorders.

### 3.4 Minerals Content of Pupa and Adult for Drones and Workers of Honeybee *A*. *Mellifera* L.

According to Table 4, potassium and sodium dominate all nine minerals in the four honeybee samples. The adult drone had the highest value of potassium (2521.4 mg/100 g), whereas the pupae drone had the highest value of sodium (685.5 mg/100 g). Nutrition specialists also recommend maintaining a potassium‐to‐sodium ratio of three to one in meals, especially for hypertensives (Kim, Yu, and Shin 2024). It's wonderful that all honeybee samples meet the K/Na ratio recommendation.

**TABLE 4:** Mineral content (mg/100 g) of pupa and adults for drones and workers of honeybee *A*. *mellifera* L.

Minerals (mg/100 g)	Drone (pupa)	Worker (pupa)	Drone (adult)	Worker (adult)
Calcium (Ca)	262.6	379.7	219.5	305.1
Magnesium (Mg)	273.0	209.7	242.3	294.7
Sodium (Na)	685.5	536.7	504.7	615.6
Potassium (K)	2028.5	2201.3	2521.4	1856.7
Iron (Fe)	10.5	12.3	24.6	20.4
Zinc (Zn)	6.4	8.7	8.8	12.6
Copper (Cu)	1.2	2.0	2.4	1.5
Manganese (Mn)	5.6	5.5	4.3	9.4
Phosphorus (P)	70.2	64.7	104.1	60.8

Ghosh, Jung, and Meyer‐Rochow (2016) found that potassium had the highest values, which were 2207.3 and 1585.4 mg/100 g for pups and workers, respectively. Ghosh et al. (2020) found potassium content was the second biggest element (after phosphorus) in Korean drone pupae and adults, which were 593.8 and 784.0 mg/100 g. In the same study, pupae and adults of Danish drones had the highest potassium content, which was 1101.98 and 1465.23 mg/100 g, respectively (Ghosh et al. 2020). Table 4 shows that drone adults have higher potassium, iron, copper, and phosphorus concentrations than the other three samples: 2521.4, 24.6, 2.4, and 104.1 mg/100 g, respectively. Sodium content was the highest level in drone pupae (685.5 mg/100 g) compared to other samples. Magnesium, zinc, and manganese were at the highest levels in adult workers, which were 294.7, 12.6, and 9.4 mg/100 g, respectively. Table 4 shows that worker pups and adults had the highest calcium levels, which were 379.7 and 305.1 mg/100 g, respectively. The body needs calcium to create and strengthen bones and teeth, as well as for bone growth and health and heart, muscle, and nerve health. In addition to bone health, calcium plus vitamin D may prevent cancer, diabetes, and high blood pressure, according to multiple studies. However, everyone needs 1000 mg of calcium every day (McGuire 2016).

According to Ghosh, Jung, and Meyer‐Rochow (2016), a third of calcium is absorbed, and sodium increases calcium release. Our findings showed that sodium was the second most abundant mineral after potassium, which may maximize calcium usage. Drone pupae and worker adults had the highest sodium levels at 685.5 and 615.6 mg/100 g (Table 4). It takes a little sodium to convey nerve impulses, contract and relax muscles, and balance water and minerals. Sodium regulates the fluid levels outside cells. These critical functions need 500 mg of salt every day. Hypertension, heart problems, and brain bleeding can result from high salt intake. It may deplete calcium and destroy bone calcium (Grillo et al. 2019). Diet reference for muscle, neuron, and energy production: the body needs magnesium in addition to sodium and potassium. Lower magnesium levels rarely cause symptoms. Low levels can induce osteoporosis, type 2 diabetes, high blood pressure, and heart disease. An irregular heartbeat or cardiac arrest, diarrhea, and nausea can develop from high magnesium levels (350 and 400 mg daily for women and men). Our investigation found magnesium levels in drone and worker phases that are human‐safe. Worker adults and drone pupae had the most magnesium at 294.7 and 273.0 mg/100 g. Our Mg values were 177.0, 118.9, and 201.7 mg/100 g, higher than Studier and Sevick (1992) and Ghosh, Jung, and Meyer‐Rochow (2016).

All tissues need potassium, sometimes called an electrolyte; its little electrical charge activates cells and neurons. Maintaining cell fluid levels is its main function. Potassium controls blood pressure and muscular contraction. Men should drink 3000 mg of potassium daily and women 2300 mg (Shrimanker and Bhattarai 2019). Furthermore, Table 4 shows that the adult honeybee drone had the greatest potassium concentration, 2521.4 mg/100 g, roughly double the values of Studier and Sevick (1992), who found 1275.0 mg/100 g for *A. mellifera* without mentioning sex. Our data found 2207.3 mg/100 g potassium in worker pupae as near as Ghosh, Jung, and Meyer‐Rochow (2016), which was 2201.3 mg/100 g. However, our adult worker potassium level was higher (1856.7 mg/100 g) than Ghosh, Jung, and Meyer‐Rochow (2016) (1585.4 mg/100 g). Potassium levels were the highest ones in drones and workers of all minerals. Honeybees contain more potassium than most edible insects (Chakravorty et al. 2014).

Iron is essential to form oxygen‐carrying hemoglobin and red blood cells. Anemia can result from iron deficiency. Daily iron intake should be 10–15 mg. Iron levels were higher for adult drones and workers (24.6 and 20.4mg/100g, respectively) than pupal stages of them (10.5 and 12.3 mg/100g, respectively). Also, Studier and Sevick (1992) and Ghosh, Jung, and Meyer‐Rochow (2016) found 37.5 and 37.7 mg/100 g for adult workers, respectively, higher than our values. According to Ghosh, Jung, and Meyer‐Rochow (2016), all honeybee worker stages had more iron than pork, veal, chicken, beef, and egg, which had 1.4, 4.3, 3.1, 2.6, and 7.3mg/100g, respectivelyt.

Zinc is an essential micromineral involved in over 300 enzymes, crucial for immunity, wound healing, and metabolism of proteins, carbohydrates, lipids, and nucleic acids. It aids in taste, scent, DNA synthesis, and gene expression, potentially preventing acne and inflammation. Daily intake: 8 mg for women, 11 mg for men. Our results showed the highest zinc levels in adult workers (12.6 mg/100 g), followed by adult drones (8.8 mg/100 g) (Table 4). These findings are consistent with Ghosh, Jung, and Meyer‐Rochow (2016), who reported a zinc level of 11.7 mg/100 g in worker pupae. The findings confirmed those of Ghosh, Jung, and Meyer‐Rochow (2016) that honeybees have more zinc than chicken, eggs, and every meat except veal.

Moderate copper intake is vital for nerve cell protection, immune support, and red blood cell and collagen production. It acts as an antioxidant, inhibiting free radicals, and is essential for cellular respiration, neurotransmitter production, pigment formation, and connective tissue strength. Safe daily intake is 1.0 mg. Table 4 shows copper had the lowest value compared to other minerals, whereas drone adults had the highest value (2.4 mg/100 g), followed by worker pupae (2.0 mg/100 g), and adult workers (1.5 mg/100 g), and then drone pupae (1.2 mg/100 g). Ghosh, Jung, and Meyer‐Rochow (2016) reported copper levels of 3.6, 3.7, and 4.6 mg/100 g in honeybee worker larvae, pupae, and adults, respectively. These copper concentrations exceed those found in eggs, chicken, and beef but remain safe for public health. Additionally, honeybee larvae contain more protein than pork and have vitamin and mineral profiles comparable to chicken and shrimp (Niijima, Matsuka, and Okada 1986; Ali et al. 2016). Also, Honeybee larvae were found to contain calcium (0.50 mg), sodium (4.40 mg), potassium (83.10 mg), magnesium (26.80 mg), iron (1.89 mg), copper (0.04 mg), zinc (1.05 mg), thiamine (0.20 mg), and riboflavin (Niijima, Matsuka, and Okada 1986). The larvae also had sodium, potassium, phosphorus, and selenium. Phosphorus and manganese play essential roles in bone formation, immune system support, ATP production, and energy storage. In this study, adult drones had the highest level of phosphorus (104.1 mg/100 g), while adult workers had the highest level of manganese (9.4 mg/100 g) (Table 4).

### 3.5 Total Phenols, Total Flavonoids, and Antioxidant Activity of Pupa and Adult for Drones and Workers of Honeybee *A*. *Mellifera* L.

The results in Table 5 showed that the drone adults had the higher amount of total phenolic content (TPC) and total flavonoids content (TFC) (9.243 mg GAE/g, 2.215 mg CE/g), followed by worker adults (5.783 mg GAE/g, 0.793 mg CE/g). Also, there were no significant differences between drones (pupae) and workers (pupae) in TPC and TFC contents. Polyphenols extracted from plants and some insects have received great interest due to their chemical diversity and interaction with other food molecules (Kosar, Dorman, and Hiltunen 2005). Therefore, studies have been conducted to evaluate the honeybee's extraction of polyphenols, including flavonoid content (Zlotek et al. 2016). Also, Table 5 represents antioxidant activity, including DPPH and FRAP methods for drone pupae and adults as well as workers pupae and adults. Adult drones exhibited the highest antioxidant activity using DPPH and FRAP, followed by adult workers, which is presumably due to the presence of high amounts of polyphenols and flavonoids. Furthermore, high amounts of amino acids like aspartic acid, glutamic acid, cysteine, and lysine can improve the consequences of oxidative stress (Duan et al. 2016).

**TABLE 5:** Total phenols, total flavonoids, and antioxidant activity of pupa and adults for drones and workers of honeybee *A*. *mellifera* L.

Sample	Total phenols (mg GAE/g)	Total flavonoids (mg CE/g)	DPPH (mg TE/g)	FRAP (mM Ferous Sulfate/g)
Drone (pupa)	5.708	0.524	3.034	5.075
Worker (pupa)	5.661	0.501	2.703	4.884
Drone (adult)	9.243	2.215	3.206	6.286
Worker (adult)	5.783	0.793	3.104	5.343

Phenolic compounds of the studied four samples of honeybees were fractionated as presented in Table 6 by HPLC. The HPLC analysis indicated that honeybee's phenolic acids consisted of different compounds (i.e., gallic acid, protocatechuic acid, p‐hydroxybenzoic acid, catechin, chlorogenic acid, caffeic acid, syringic acid, quercetin, and kaempferol acid) at different concentrations. Moreover, p‐hydroxybenzoic acid was the predominant phenolic acid in all honeybee samples. The results showed that adult drones had the highest value (20095.72 μg/100 g) of most fractionated phenolic compounds, followed by adult workers (13151.85 μg/100 g). In addition, adult drones contained the highest levels of p‐hdroxybenzoic acid, gallic acid, chlorogenic acid, caffeic acid, and quercetin. From the tabulated data, it was found that adults of both drones and workers were an excellent source of the most phenolic compounds. Additionally, pupae drone samples had a good and large value (10561.85 μg/100 g) compared to pupae workers (8007.16 μg/100 g).

**TABLE 6:** Phenolic compounds (μg/100 g) of pupa and adults for drones and workers of honeybee *A. mellifera* L.

Compound (μg/100 g)	Drone (pupa)	Worker (pupa)	Drone (adult)	Worker (dult)
Gallic acid	0.000	0.000	1061.178	0.000
Protocatechuic acid	323.158	197.807	901.645	255.713
p‐hydroxybenzoic acid	8056.248	7294.573	15478.353	6558.392
Catechin	1561.993	0.000	1441.993	683.056
Chlorogenic acid	263.154	167.474	899.016	155.579
Caffeic acid	0.000	0.000	113.473	0.000
Syringic acid	357.023	347.306	0.000	0.000
Quercetin	0.000	0.000	200.058	1790.501
Kaempferol	0.000	0.000	0.000	3708.613
Total	10561.58	8007.16	20095.72	13151.85

Table 7 presents the color values (L*, a*, and b*) of drone and worker pupae and adults of the honeybee *Apis mellifera* L. The L* value represents lightness, where higher values indicate lighter coloration, while the a* and b* values represent the red‐green and yellow‐blue color axes, respectively. The rise of the L* value shows the lightness of each sample, which was ordered as follows: Drone (pupa) > Worker (pupa) > Drone (adult) > Worker (adult). The lightness increased with increasing L* value and decreased with decreasing L* value. Also, there were no big differences among the a* values of Drone (pupa), Worker (pupa), and Drone (adult), while it was significant with Worker (adult). Moreover, b* values were ordered as follows: Drone (pupa) > Worker (pupa) > Drone (adult) > Worker (adult) as in L*values.

**TABLE 7:** Color values (L*, a*, and b*) of pupa and adults for drones and workers of honeybee *A. mellifera* L.

Treatments	L*	a*	b*
Drone (pupa)	30.87 ± 0.24a	5.06 ± 0.10b	15.28 ± 0.10a
Worker (pupa)	23.63 ± 0.17b	6.29 ± 0.16a	12.11 ± 0.15b
Drone (adult)	13.47 ± 0.26c	5.75 ± 0.11ab	8.76 ± 0.13c
Worker (adult)	6.40 ± 0.31d	2.92 ± 0.14c	3.60 ± 0.09d

*Note:* The experimental values (means and SD for *n* = 3) with small letters are significantly different (*p* ≤ 0.05).

### 3.6 Cytotoxic Effect on Human Cell Lines for Pupa and Adult of Drones and Workers

**FIGURE 1:**
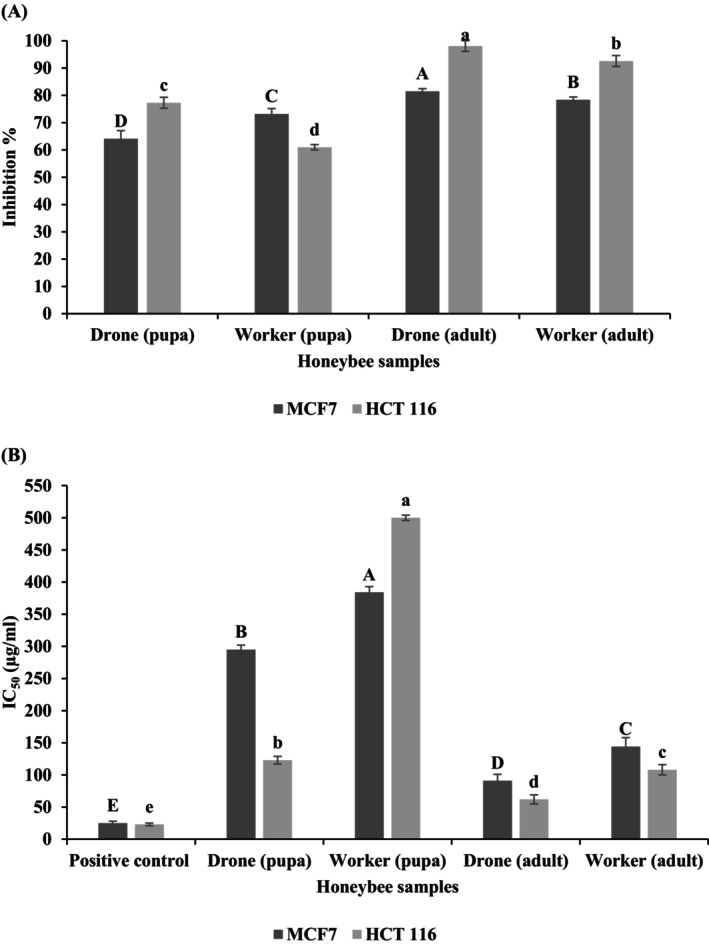
Anticancer effect of honeybee samples (drone pupae, worker pupae, drone adult, and worker adult) against MCF_7_ and HCT_116_ cell lines [(A) inhibition%; (B) IC_50_]. IC_50_, Lethal concentration of the sample which causes the death of 50% of cells in 48 h; MCF_7_, Human Caucasian breast adenocarcinoma; HCT_116_, Human colorectal carcinoma cell line; Positive control Adriamycin (Doxorubicin). (a–d) different superscripts in a column were significantly different (*p* < 0.05); data are means ± SD (*n* = 3).

In order to evaluate the anticancer properties of the honeybee samples, two types of cancer cells (MCF7, Human Caucasian breast adenocarcinoma; HCT116, Human colorectal carcinoma) were exposed to samples derived from drone pupae, worker pupae, drone adults, and worker adults. The results were compared to those achieved with the conventional medication, vinblastine sulfate. Based on the data presented in Figure 1, the powder of adult drone showed the most potent inhibition of MCF_7_ and HCT_116_ cells, with effectiveness levels of 98.1% and 81.5%, respectively. This is because it has a greater concentration of total phenolic compounds, amino acids, and antioxidant values compared to other honeybee samples. Furthermore, a clear relationship was seen between values and the inhibition of cancer cells, specifically breast and human colorectal carcinoma malignancy, as indicated in Tables 5 and 6. Several studies have already documented the anticancer properties of phenolic substances. The primary reason for this impact was the existence of antioxidant chemicals, particularly Total Phenolic Content (TPC) and Total Flavonoid Content (TFC) (Owen et al. 2000). The findings of our study were consistent with those of Wieczynska et al. (2017), suggesting a notable decrease in mitochondrial function, which could have led to a decrease in the overall health of the cells.

IC_50_ is the concentration of a sample that causes the death of 50% of cells within 48 h. In this study, an inverse relationship between inhibition percentage and IC_50_ was observed (Figure 1A,B). The positive control was doxorubicin, an anticancer drug for hematologic and solid tumors. Among the samples tested, the lowest IC_50_ values were observed for the drone adult sample (91 μg/mL and 62 μg/mL for MCF_7_ and HCT_116_ cells, respectively) as the best response, followed by the worker adult sample (144 μg/mL and 108 μg/mL for MCF_7_ and HCT_116_ cells, respectively). Also, the third IC_50_ sample was drone pupae (295 μg/mL, 123 μg/mL for MCF_7_ and HCT_116_ cells, respectively). Finally, the last ordered sample indicated the highest IC_50_ with the lowest inhibition % was observed in the worker pupae sample (384 μg/mL and 500 μg/mL for MCF_7_ and HCT_116_ cells, respectively), as depicted in Figure 1B. The best values of antioxidant % and anticancer activity (inhibition % and IC_50_) were recorded in the drone adult sample, which can be attributable to its phenolic components and flavonoid content, as indicated in Tables 5 and 6.

## 4 Conclusion

In conclusion, this study explored the nutritional composition and bioactive properties of honeybee pupa and adult stages, focusing on drones and worker bees. Drones exhibited higher protein, fat, amino acids (excluding tryptophan), fatty acids (especially oleic and palmitic), potassium, sodium, and phenolic compounds than worker bees. These findings suggest the potential benefits of bee (especially adult drones) products as functional foods or supplements. Additionally, the cytotoxic effect against breast and colorectal cancer cells highlights their potential for therapeutic applications. Further research is warranted to fully understand and harness the health‐promoting properties of honeybee products for human consumption and medical purposes.

## Author Contributions

**Tarek Gamal Abedelmaksoud:** conceptualization (equal), data curation (equal), formal analysis (equal), funding acquisition (equal), investigation (equal), methodology (equal), project administration (equal), software (equal), writing – original draft (equal), writing – review and editing (equal). **Mohamed Salah El‐Masarawy:** conceptualization (equal), data curation (equal), formal analysis (equal), investigation (equal), methodology (equal), software (equal), writing – original draft (equal). **Ammar B. Altemimi:** conceptualization (equal), software (equal), writing – review and editing (equal). **Mohammad Ali Hesarinejad:** conceptualization (equal), software (equal), writing – review and editing (equal). **Ahmed M. S. Hussein:** conceptualization (equal), investigation (equal), methodology (equal), writing – original draft (equal). **Sayed Saad Smuda:** conceptualization (equal), investigation (equal), methodology (equal), writing – original draft (equal)

## Ethics Statement

The authors have nothing to report.

## Consent

All authors have read and agreed to the published version of the manuscript. All authors read and approved the final manuscript.

## Conflicts of Interest

The authors declare no conflicts of interest.

## Data Availability

All data generated or analyzed during this study are included in this published article.

## References

[fsn34581-bib-0001] Abedelmaksoud, T. G. , S. M. Mohsen , L. Duedahl‐Olesen , M. M. Elnikeety , and A. H. Feyissa . 2018. “Optimization of Ohmic Heating Parameters for Polyphenoloxidase Inactivation in Not‐From‐Concentrate Elstar Apple Juice Using RSM.” Journal of Food Science and Technology 55: 2420–2428. 30042557 10.1007/s13197-018-3159-1 PMC6033792

[fsn34581-bib-0057] Açikgöz, Z. , and B. Yücel . 2016. “Using Facilities of Apilarnil (Bee Drone Larvae) in Poultry Nutrition.” Godina LXI Broj 61: 12–15. 10.1016/156.208.117.59. CABI Record Number: 20163288151.

[fsn34581-bib-0002] Ali, I. , S. Zhang , J. Y. Luo , C. Y. Wang , L. M. Lv , and J. J. Cui . 2016. “Artificial Diet Development and Its Effect on the Reproductive Performances of Propylea Japonica and Harmonia Axyridis.” Journal of Asia‐Pacific Entomology 19, no. 2: 289–293.

[fsn34581-bib-0003] AOAC International . 2016. Official Methods of Analysis of AOAC International. 20th ed. Berlin, Germany: AOAC International.

[fsn34581-bib-0004] Benzie, I. F. , and S. W. Choi . 2014. “Antioxidants in Food: Content, Measurement, Significance, Action, Cautions, Caveats, and Research Needs.” Advances in Food and Nutrition Research 71: 1–53. 10.1016/B978-0-12-800270-4.00001-8 24484938

[fsn34581-bib-0005] Bogdanov, S. 2011. “Royal Jelly, Bee Brood: Composition, Health, Medicine: A Review.” Lipids 3, no. 8: 8–19.

[fsn34581-bib-0006] Chakravorty, J. , S. Ghosh , C. Jung , and V. B. Meyer‐Rochow . 2014. “Nutritional Composition of Chondacris Rosea and Brachytrupes Orientalis: Two Common Insects Used as Food by Tribes of Arunachal Pradesh, India.” Journal of Asia‐Pacific Entomology 17, no. 3: 407–415. 10.1016/j.aspen.2014.03.007

[fsn34581-bib-0007] Choi, J. S. 2021. “Nutrition, Safety, Health Functional Effects, and Availability of Honeybee (*Apis mellifera* L.) Drone Pupae.” Insects 12: 771. 10.3390/insects12090771 34564210 PMC8468450

[fsn34581-bib-0008] Duan, J. , J. Yin , W. Ren , et al. 2016. “Dietary Supplementation With l‐Glutamate and l‐Aspartate Alleviates Oxidative Stress in Weaned Piglets Challenged With Hydrogen Peroxide.” Amino Acids 48, no. 1: 53–64. 10.1007/s00726-015-2065-3 26255283

[fsn34581-bib-0009] EFSA Scientific Committee . 2015. “Risk Profile Related to Production and Consumption of Insects as Food and Feed.” EFSA Journal 13: 4257.

[fsn34581-bib-0010] Ekpo, K. E. , and A. O. Onigbinde . 2005. “Nutritional Potentials of the Larva of Rhynchophorus Phoenicis (F).” Pakistan Journal of Nutrition 4, no. 5: 287–290. 10.3923/pjn.2005.287.290

[fsn34581-bib-0011] El‐Masarawy, M. S. , H. M. El‐Bendary , and A. M. A. El‐Helaly . 2021. “The Effect of Using Imidacloprid and Chlorpyrifos and Their Nanoforms on Certain Characteristics of Honeybee *Apis mellifera* L.” International Journal of Tropical Insect Science 41: 1037–1042. 10.1007/s42690-020-00286-6

[fsn34581-bib-0012] El‐Menshawi, B. S. , W. Fayad , K. Mahmoud , S. M. El‐Hallouty , and M. El‐Manawaty . 2010. “Screening of Natural Products for Therapeutic Activity Against Solid Tumors.” Indian Journal of Experimental Biology 48, no. 3: 258–264. 21046978

[fsn34581-bib-0013] Elsayed, N. , H. S. El‐Din , A. B. Altemimi , H. Y. Ahmed , A. Pratap‐Singh , and T. G. Abedelmaksoud . 2021. “In Vitro Antimicrobial, Antioxidant and Anticancer Activities of Egyptian Citrus Beebread.” Molecules 26, no. 9: 2433. 10.3390/molecules26092433 33922031 PMC8122611

[fsn34581-bib-0014] Elsayed, N. , D. A. Marrez , M. A. Ali , A. A. A. El‐Maksoud , W. Cheng , and T. G. Abedelmaksoud . 2022. “Phenolic Profiling and In‐Vitro Bioactivities of Corn (*Zea mays* L.) Tassel Extracts by Combining Enzyme‐Assisted Extraction.” Food 11, no. 14: 2145. 10.3390/foods11142145 PMC9320485 35885388

[fsn34581-bib-0015] Finke, M. D. 2005. “Nutrient Composition of Bee Brood and Its Potential as Human Food.” Ecology of Food and Nutrition 44, no. 4: 257–270. 10.1080/03670240500187278

[fsn34581-bib-0016] Friedt, W. , and R. Snowdon . 2010. “Oilseed rape.” Oil Crops 20: 91–126.

[fsn34581-bib-0017] Ganogpichayagrai, A. , and C. Suksaard . 2020. “Proximate Composition, Vitamin and Mineral Composition, Antioxidant Capacity, and Anticancer Activity of Acanthopanax Trifoliatus.” Journal of Advanced Pharmaceutical Technology & Research 11, no. 4: 179–183. 33425701 10.4103/japtr.JAPTR_61_20 PMC7784940

[fsn34581-bib-0018] Ghaly, A. E. , and F. N. Alkoaik . 2009. “The Yellow Mealworm as a Novel Source of Protein.” American Journal of Agricultural and Biological Sciences 4: 319–331. 10.3844/ajabssp.2009.319.331

[fsn34581-bib-0019] Ghaly, A. E. , and F. N. Alkoaik . 2010. “Nutritional Value of the Maize Stalk Borer and American Bollworm as Unconventional Protein Sources.” American Journal of Applied Sciences 7: 1–12. 10.3844/AJASSP.2010.1.12

[fsn34581-bib-0020] Ghosh, S. , C. Jung , and V. B. Meyer‐Rochow . 2016. “Nutritional Value and Chemical Composition of Larvae, Pupae, and Adults of Worker Honey Bee, Apis Mellifera Ligustica as a Sustainable Food Source.” Journal of Asia‐Pacific Entomology 19: 487–495. 10.1016/j.aspen.2016.03.008

[fsn34581-bib-0021] Ghosh, S. , H. Y. Sohn , S. J. Pyo , A. B. Jensen , V. B. Meyer‐Rochow , and C. Jung . 2020. “Nutritional Composition of Apis Mellifera Drones From Korea and Denmark as a Potential Sustainable Alternative Food Source: Comparison Between Developmental Stages.” Food 9: 389. 10.3390/foods9040389 PMC7230812 32230865

[fsn34581-bib-0022] Grillo, A. , L. Salvi , P. Coruzzi , P. Salvi , and G. Parati . 2019. “Sodium Intake and Hypertension.” Nutrients 11, no. 9: 1970. 10.3390/nu11091970 31438636 PMC6770596

[fsn34581-bib-0023] Hu, F. , and Y. Li . 2001. “Nutritive value and pharmacological actions of Italian worker bee larvae and pupae.” Proceedings of the 37th International Apicultural Congrress, 28 October–1 November 2001, Durban South Africa: produced by: Document Transformation Technologies. https://www.apimondia.com/docs/congress/2001/papers/249.pdf

[fsn34581-bib-0024] Isidorov, V. A. , S. Bakier , and M. Stocki . 2016. “GC‐MS Investigation of the Chemical Composition of Honeybee Drone and Queen Larvae Homogenate.” Journal of Apicultural Science 60: 111–120. 10.1515/jas-2016-0011

[fsn34581-bib-0025] Jensen, A. B. , J. Evans , A. Jonas‐Levi , et al. 2019. “Standard Methods for Apis Mellifera Brood as Human Food.” Journal of Apicultural Research 58: 1–28. 10.1080/00218839.2016.1226606

[fsn34581-bib-0026] Kim, B. S. , M. Y. Yu , and J. Shin . 2024. “Effect of Low Sodium and High Potassium Diet on Lowering Blood Pressure and Cardiovascular Events.” Clinical Hypertension 30, no. 1: 2. 38163867 10.1186/s40885-023-00259-0 PMC10759559

[fsn34581-bib-0027] Kim, K. H. , R. Tsao , R. Yang , and S. W. Cui . 2006. “Phenolic Acid Profiles and Antioxidant Activities of Wheat Bran Extracts and the Effect of Hydrolysis Conditions.” Food Chemistry 95: 466–473. 10.1016/j.foodchem.2005.01.032

[fsn34581-bib-0028] Kim, S. G. , S. O. Woo , K. W. Bang , H. R. Jang , and S. M. Han . 2018. “Chemical Composition of Drone Pupa of Apis Mellifera and Its Nutritional Evaluation.” Korean Journal of Apiculture 33: 17–23. 10.17519/apiculture.2018.04.33.1.17

[fsn34581-bib-0029] Kosar, M. , H. J. D. Dorman , and R. Hiltunen . 2005. “Effect of an Acid Treatment on the Phytochemical and Antioxidant Characteristics of Extracts From Selected Lamiaceae Species.” Food Chemistry 91: 525–533. 10.1016/j.foodchem.2004.06.029

[fsn34581-bib-0030] Lazaryan, D. S. 2002. “Comparative Amino Acid Analysis of Bee Brood.” Pharmaceutical Chemistry Journal 36: 680–682. 10.1023/A:1023469931357

[fsn34581-bib-0031] Lazaryan, D. S. , and E. M. Sotnikova . 2004. “Determination of the Content of Lipid Phosphorus and Phospholipids in Bee Brood.” Pharmaceutical Chemistry Journal 38: 517–519.

[fsn34581-bib-0032] Li, M. , C. Mao , X. Li , et al. 2023. “Edible Insects: A New Sustainable Nutritional Resource Worth Promoting.” Food 12, no. 22: 4073. 10.3390/foods12224073 PMC10670618 38002131

[fsn34581-bib-0033] Makkar, H. P. , G. Tran , V. Heuzé , and P. Ankers . 2014. “State‐Of‐The‐Art on Use of Insects as Animal Feed.” Animal Feed Science and Technology 197: 1–33.

[fsn34581-bib-0034] McGuire, S. 2016. “Scientific Report of the 2015 Dietary Guidelines Advisory Committee.” Advances in Nutrition 7: 202–204. 26773024 10.3945/an.115.011684 PMC4717899

[fsn34581-bib-0035] Mensink, R. , and M. Katan . 1987. “Effect of Monounsaturated Fatty Acids Versus Complex Carbohydrates on High‐Density Lipoproteins in Healthy Men and Women.” Lancet 329: 122–125. 10.1016/s0140-6736(87)91965-9 2879969

[fsn34581-bib-0036] Mensink, R. P. 1993. “Effects of the Individual Saturated Fatty Acids on Serum Lipids and Lipoprotein Concentrations.” American Journal of Clinical Nutrition 57: 711–714. 10.1093/ajcn/57.5.711s 8475888

[fsn34581-bib-0037] Mensink, R. P. 2005. “Effects of Stearic Acid on Plasma Lipid and Lipoproteins in Humans.” Lipids 40: 1201–1205. 10.1007/s11745-005-1486-x 16477803

[fsn34581-bib-0038] Mohamed, R. M. , M. R. Ali , S. S. Smuda , and T. G. Abedelmaksoud . 2021. “Utilization of Sugarcane Bagasse Aqueous Extract as a Natural Preservative to Extend the Shelf Life of Refrigerated Fresh Meat.” Brazilian Journal of Food Technology 24: e2020167.

[fsn34581-bib-0039] Mohsen, S. M. , A. Ashraf , S. S. Ahmed , and T. G. Abedelmaksoud . 2024. “Biscuits Enriched With the Edible Powder of Angoumois Grain Moth (*Sitotroga cerealella*): Optimization, Characterization and Consumer Perception Assessment.” Food Systems 7, no. 1: 165–178.

[fsn34581-bib-0040] Niijima, K. , M. Matsuka , and I. Okada . 1986. “Artificial Diets for an Aphidophagous Coccinellid Harmonia Axyridis and Its Nutrition.” Ecology of Aphidophaga 8: 37–50.

[fsn34581-bib-0056] Nowak, V. , D. Persijn , D. Rittenschober , and U. R. Charrondiere . 2016. “Review of Food Composition Data for Edible Insects.” Food Chemistry 193: 39–46. 10.1016/j.foodchem.2014.10.114 26433285

[fsn34581-bib-0041] Owen, R. W. , A. Giacosa , W. E. Hull , R. Haubner , B. Spiegelhalder , and H. Bartsch . 2000. “The Antioxidant/Anticancer Potential of Phenolic Compounds Isolated From Olive Oil.” European Journal of Cancer 36: 1235–1247. 10.1016/s0959-8049(00)00103-9 10882862

[fsn34581-bib-0042] Poma, G. , M. Cuykx , E. Amato , C. Calaprice , J. F. Focant , and A. Covaci . 2017. “Evaluation of Hazardous Chemicals in Edible Insects and Insect‐Based Food Intended for Human Consumption.” Food and Chemical Toxicology 100: 70–79. 28007452 10.1016/j.fct.2016.12.006

[fsn34581-bib-0043] Raheem, D. , C. Carrascosa , O. B. Oluwole , et al. 2019. “Traditional Consumption of and Rearing Edible Insects in Africa, Asia and Europe.” Critical Reviews in Food Science and Nutrition 59, no. 14: 2169–2188. 29446643 10.1080/10408398.2018.1440191

[fsn34581-bib-0044] Ramos‐Elorduy, J. 2005. “Insects: A Hopeful Food Source.” Ecological Implications of Minilivestock 20: 263–291.

[fsn34581-bib-0045] Rutka, I. , R. Galoburda , J. Galins , and A. Galins . 2021. “Bee Drone Brood Homogenate Chemical Composition, Stabilization and Application: A Review.” Research for Rural Development 36: 96–103. 10.22616/rrd.27.2021.014

[fsn34581-bib-0046] Sawczuk, R. , J. Karpinska , D. Filipowska , A. Bajguz , and M. Hryniewicka . 2022. “Evaluation of Total Phenols Content, Anti‐DPPH Activity and the Content of Selected Antioxidants in the Honeybee Drone Brood Homogenate.” Food Chemistry 368: 130745. 34404004 10.1016/j.foodchem.2021.130745

[fsn34581-bib-0047] Shrimanker, I. , and S. Bhattarai . 2019. “Electrolytes.” https://www.altmetric.com/details/85104175 31082167

[fsn34581-bib-0048] Smith, P. , K. Calvin , J. Nkem , et al. 2020. “Which Practices Co‐Deliver Food Security, Climate Change Mitigation and Adaptation, and Combat Land Degradation and Desertification?” Global Change Biology 26, no. 3: 1532–1575. 31637793 10.1111/gcb.14878 PMC7079138

[fsn34581-bib-0049] Studier, E. H. , and S. H. Sevick . 1992. “Live Mass, Water Content, Nitrogen and Mineral Levels in Some Insects From South‐Central Lower Michigan.” Comparative Biochemistry and Physiology Part A: Physiology 103: 579–595. 10.1016/0300-9629(92)90293-Y

[fsn34581-bib-0050] Talwar, R. , M. Freymond , K. Beesabathuni , and S. Lingala . 2024. “Current and Future Market Opportunities for Alternative Proteins in Low‐and Middle‐Income Countries.” Current Developments in Nutrition 8: 102035. 38476721 10.1016/j.cdnut.2023.102035 PMC10926118

[fsn34581-bib-0051] Wehbe, R. , J. Frangieh , M. Rima , D. El Obeid , J. M. Sabatier , and Z. Fajloun . 2019. “Bee Venom: Overview of Main Compounds and Bioactivities for Therapeutic Interests.” Molecules 24, no. 16: 2997. 31430861 10.3390/molecules24162997 PMC6720840

[fsn34581-bib-0052] Wieczynska, A. , J. Wezgowiec , W. Wieckiewicz , et al. 2017. “Antimicrobial Activity, Cytotoxicity and Total Phenolic Content of Different Extracts of Propolis From the West Pomeranian Region in Poland.” Acta Poloniae Pharmaceutica 74: 715–722. 29624279

[fsn34581-bib-0053] Yen, A. L. 2009. “Edible Insects: Traditional Knowledge or Western Phobia?” Entomological Research 39, no. 5: 289–298.

[fsn34581-bib-0054] Zahran, H. A. , and H. Z. Tawfeuk . 2019. “Physicochemical Properties of New Peanut (*Arachis hypogaea* L.) Varieties.” Oilseeds & Fats, Crops and Lipids 26: 19–25. 10.1051/ocl/2019018

[fsn34581-bib-0055] Zlotek, U. , S. Mikulska , M. Nagajek , and M. Świeca . 2016. “The Effect of Different Solvents and Number of Extraction Steps on the Polyphenol Content and Antioxidant Capacity of Basil Leaves (*Ocimum basilicum* L.) Extracts.” Saudi Journal of Biological Sciences 23: 628–633. 10.1016/j.sjbs.2015.08.002 27579013 PMC4992113

